# Sensory capability and information integration independently explain the cognitive status of healthy older adults

**DOI:** 10.1038/s41598-020-80069-8

**Published:** 2020-12-31

**Authors:** Jonas Misselhorn, Florian Göschl, Focko L. Higgen, Friedhelm C. Hummel, Christian Gerloff, Andreas K. Engel

**Affiliations:** 1 Department of Neurophysiology and Pathophysiology, University Medical Center Hamburg-Eppendorf, 20246 Hamburg, Germany; 2 Department of Neurology, University Medical Center Hamburg-Eppendorf, 20246 Hamburg, Germany; 3 Defitech Chair of Clinical Neuroengineering, Center for Neuroprosthetics and Brain Mind Institute, Swiss Federal Institute of Technology (EPFL), Geneva, Switzerland; 4 Defitech Chair of Clinical Neuroengineering, Center for Neuroprosthetics and Brain Mind Institute, Swiss Federal Institute of Technology Valais (EPFL Valais), Clinique Romande de Réadaptation, Sion, Switzerland; 5 Clinical Neuroscience, Medical School University of Geneva, Geneva, Switzerland

**Keywords:** Cognitive ageing, Sensory processing

## Abstract

While there is evidence that sensory processing and multisensory integration change with age, links between these alterations and their relation to cognitive status remain unclear. In this study, we assessed sensory thresholds and performance of healthy younger and older adults in a visuotactile delayed match-to-sample task. Using Bayesian structural equation modelling (BSEM), we explored the factors explaining cognitive status in the group of older adults. Additionally, we applied transcranial alternating current stimulation (tACS) to a parieto-central network found to underlie visuotactile interactions and working memory matching in our previous work. Response times and signal detection measures indicated enhanced multisensory integration and enhanced benefit from successful working memory matching in older adults. Further, tACS caused a frequency-specific speeding (20 Hz) and delaying (70 Hz) of responses. Data exploration suggested distinct underlying factors for sensory acuity and sensitivity d’ on the one side, and multisensory and working memory enhancement on the other side. Finally, BSEM showed that these two factors labelled ‘sensory capability’ and ‘information integration’ independently explained cognitive status. We conclude that sensory decline and enhanced information integration might relate to distinct processes of ageing and discuss a potential role of the parietal cortex in mediating augmented integration in older adults.

## Introduction

Ageing is associated with marked changes in sensory and cognitive abilities due to alterations of the peripheral and central nervous system. Most detrimental to independence and quality of daily life is a decline in ‘higher-level’ cognitive functions^1^, such as attention^2^ and memory^3^. While a number of theories on cognitive ageing focus on changes in frontal cortices^4–6^, an intriguing hypothesis proposes that decline in sensory capabilities might mediate cognitive decline^7^. This idea is based on the established finding that simple measures of sensory acuity reliably predict cognitive abilities in older adults^7–9^. Another well-documented observation is that older adults show altered processing of crossmodal stimuli indicative of enhanced multisensory integration (‘*crossmodal*’ here referring to stimulus properties and ‘*multisensory*’ to behavioural or underlying neurophysiological processes^10^)^11,12^. Reconciling both reports of behavioural benefits and costs of multisensory integration in ageing, de Dieuleveult and colleagues recently suggested that older adults might fail to properly weigh sensory inputs according to their behavioural relevance^13^. It is an open question how changes in sensory capability and multisensory integration interact in late adulthood. Specifically, it is possible that enhanced multisensory integration is a consequence of degraded sensory processing compatible with the law of inverse effectiveness^14,15^. Alternatively, sensory capability and the tendency to integrate information across the senses might be independent and, thus, point towards distinct processes of brain ageing.

**Figure 1:**
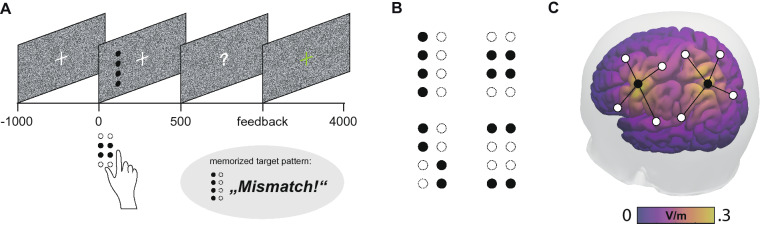
Experimental design. (**A**) Trial sequence. Participants fixated a central cross. In two thirds of all trials, tactile pattern presentation to the right index finger was accompanied by a visual dot pattern appearing on the left of the fixation cross. Participants were instructed to attend to the tactile input only, compare the tactile pattern with a blockwise-defined target pattern and report match or mismatch. (**B**) Set of dot patterns that were presented on a Braille stimulator and as a visual pattern on the screen. (**C**) tACS was applied using Ag/AgCl ring electrodes in two separate montages, centered on S1 (primary somatosensory cortex) and IPS (intraparietal sulcus) of the left hemisphere.

In the current study, we assessed sensory capability and multisensory interactions in healthy younger and older adults and modelled how these factors influence cognitive status. Our task was a variant of a well-established visuotactile congruency task^16–20^ in which participants identified tactile dot patterns, similar to Braille letters, and matched them with a target pattern held in working memory (Fig. 1A,B). In one third of the trials, tactile stimuli were presented unimodally, whereas two thirds featured additional task-irrelevant visual input that could either be congruent or incongruent to the tactile stimulus. Building on previous findings^20–23^, we hypothesized, first, that older adults would profit more from redundant but congruent visual input in tactile target detection. According to the idea of improper sensory weighing in later adulthood, we also expected to find enhanced behavioural costs of processing incongruent crossmodal information in older compared to younger adults. Both directions of effects would support the idea of enhanced multisensory integration with ageing^13^. Second, we investigated potential dependencies between multisensory integration, sensory capability and cognitive status. Therefore, preceding the experiment, we estimated unimodal visual and tactile thresholds and participants underwent neurological examination including cognitive assessment. Third, we modulated neurophysiological processes potentially underlying task performance by means of non-invasive brain stimulation. A candidate brain structure that could mediate age-related changes in cognitive as well as perceptual processes is the parietal cortex. Especially the posterior parietal cortex has repeatedly been found integral to both multisensory integration^24,25^ and cognitive functions^26^. Consistent with these reports, we recently described a left-hemispheric parieto-central beta-frequency (~ 20 Hz) network mediating multisensory interactions and working memory matching in an electroencephalography (EEG) study employing a comparable experimental paradigm in younger adults^19^. In order to address the role of the parietal cortex in mediating multisensory integration and its potential link to cognitive status in this study, we used bifocal transcranial alternating current stimulation (tACS) and targeted the beta-network found earlier (Fig. 1C). We expected that beta-frequency stimulation (20 Hz) would enhance stimulus processing and working memory matching, eventually leading to improved detection performance. We controlled tACS effects of beta stimulation with a sham condition as well as with an active control condition (70 Hz). Since tACS has been rarely used in older populations^27^, and the montage was designed based on neurophysiological data from younger adults, potential age-related differences in tACS susceptibility were open to exploration.

## Results

### Assessment

Preceding the experiment, participants underwent an assessment procedure consisting of a neurological examination, the 2-point-discrimination test^28^ to assess peripheral somatosensation and a test of visual acuity (Snellen chart)^29^. If necessary, the assessment as well as the experimental procedure were performed with corrected vision. Cognitive status was assessed with the Mini-Mental State Examination (MMSE)^30^ and the DemTect^31^. Using a visual analogue scale (VAS), participants further self-assessed attention level and fatigue. In the following, we report results of this assessment complemented by tactile and visual perception thresholds for the stimuli used in our task. These thresholds were estimated in a preceding study^20^ using an adaptive staircase procedure (see “Methods” for details). The group comparison of the assessment data (Table 1) showed significant differences between younger and older participants (Pillai’s Trace = 0.90, $F_{1,31}$ = 20.78, *p* < 0.001). Post-hoc comparisons showed that the two groups differed significantly in tactile ($F_{1,31}$ = 62.34, *p* < 0.001) and visual ($F_{1,31}$ = 139.41, *p* < 0.001) thresholds. Furthermore, there was a significant difference in years of education ($F_{1,31}$ = 14.04, *p* = 0.005) and in visual acuity ($F_{1,31}$ = 130.27, *p* < 0.001), but no difference in 2-point discrimination ($F_{1,31}$ = 0.42, *p* = 1). There were no significant differences in MMSE ($F_{1,31}$ = 0.56, *p* = 1) and DemTect ($F_{1,31}$ = 3.58, *p* = 0.332).

**Table 1:** Assessment data of the groups.

Metric	Younger group (n = 17)	Older group (n = 16)
Age (years)	24.4 (± 3.1)	72.6 (± 4.6)
Gender	10 females (7 males)	10 females (6 males)
Education (years)**	12.41 (± 0.62)	10.81 (± 1.64)
Tactile threshold (pin height in mm)***	0.55 (± 0.18)	1.21 (± 0.29)
Visual threshold (grey intensity in RGB)***	48.1 (± 1.3)	55.8 (± 2.3)
Visual acuity (arcmin)***	0.97 (± 0.1)	0.68 (± 0.1)
2-point-discrimination (mm)	2.06 (± 0.2)	2.13 (± 0.3)
MMSE	29.7 (± 0.5)	29.5 (± 1.0)
DemTect	17.4 (± 1.5)	16.3 (± 1.7)
Attention level (VAS in cm)	1.5 (± 1.2)	2.0 (± 1.6)
Fatigue (VAS in cm)	1.8 (± 1.3)	1.9 (± 1.7)
Fatigue of hand (VAS in cm)	0.6 (± 0.6)	1.3 (± 1.3)

Mean values are shown ± standard deviation. Based on significant main effects, post-hoc tests were conducted. Asterisks indicate significant differences between younger and older participants (**p < 0.01, ***p < 0.001).

### Behaviour

Both detection accuracies and response times were analysed according to a mixed repeated- measures ANOVA design with the following factors: AGE (between-participants, younger/older), STIMULUS (within-participants, unimodal/congruent/incongruent), TARGET (within-participants, target/non-target; only used for response times, see below) and tACS (within-participants, sham/beta/gamma). First, we report detection accuracies using signal detection theory (SDT) measures d’ and c^32,33^. Second, we present the results of a distribution-level analysis of response times (RTs) based on cumulative distribution functions (CDFs)^34^. Concluding, we show the results of Bayesian structural equation modelling (BSEM) to explain cognitive status in older adults based on compound measures of sensory capability and information integration.

### Detection accuracies

**Figure 2:**
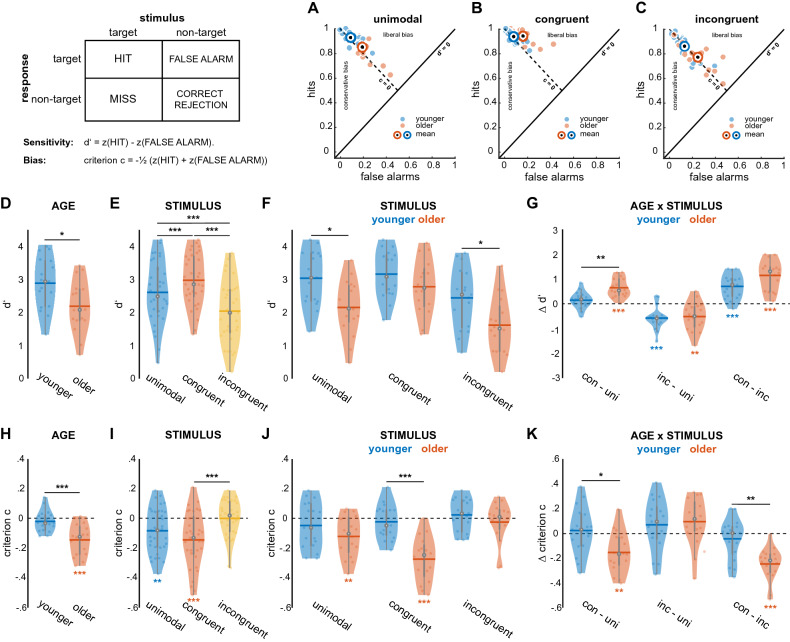
Age- and stimulus-related differences in sensitivity d′ and bias c. Violin plots show mean value (horizontal line), median (white dot), interquartile range (horizontal line) and data of individual participants (coloured dots). Black asterisks indicate statistical significance of t-tests for pairwise comparisons, coloured asterisks indicate significance for one-sample t-tests against zero (*p < 0.05, **p < 0.01, ***p < 0.001). (**A–C**) Hits and false alarms as a function of STIMULUS, separately for younger and older groups. (**D**) Effect of AGE on d′. (**E**) Effect of STIMULUS on d′. (**F**) and (**G**) Interaction between AGE and STIMULUS for d′ scores is shown as difference between conditions of STIMULUS, separately for younger and older groups. (**H**) Effect of AGE on criterion c. (**I**) Effect of STIMULUS on criterion c. (**J**) and (**K**) Interaction between AGE and STIMULUS for criterion c. All conditions of STIMULUS are shown separately for younger and older groups.

To describe the accuracy of responses in our visuotactile task, we used SDT measures sensitivity d′ and criterion location c as a metric of response bias. While d’ captures the perceiver’s ability to discriminate different choice alternatives and is calculated from hits (in our case correctly identified targets) and false alarms (1—correctly identified non-targets), bias c describes the perceiver’s tendency to categorize stimuli as either the one or the other, irrespective of actual stimulus presence (see Fig. 2, and “Methods” for details). D′ and c were subjected to a mixed repeated-measures ANOVA containing the factors AGE, STIMULUS, and tACS. In contrast to the RT analysis, the factor TARGET is missing from the analysis due to the way sensitivity measure d′ and bias c are calculated (see “Methods” and Fig. 2). In the following, all ANOVA *p*-values are reported using Greenhouse–Geisser correction. Post-hoc analysis was done using (unpaired or paired) t-tests applying Bonferroni correction for multiple comparisons if appropriate. If not stated otherwise, all effects are reported significant at *p* < 0.05. In addition, we computed effect sizes as indicated by partial eta squared for ANOVAs ($\eta_{p}^{2}$), and Cohen’s *d* for pairwise comparisons.

For sensitivity measure d′, a main effect of AGE ($F_{1,31}$ = 6.44, *p* = 0.016, $\eta_{p}^{2}$ = 0.17; see Fig. 2D) was observed with younger adults showing significantly better overall performance (2.89 ± 0.79 vs. 2.19 ± 0.79, mean ± standard deviation [SD]). In addition, we found a main effect of STIMULUS ($F_{2,62}$ = 62.61, *p* < 0.001, $\eta_{p}^{2}$ = 0.67; Fig. 2E) as well as an interaction of AGE and STIMULUS ($F_{2,62}$ = 5.28, *p* = 0.012, $\eta_{p}^{2}$ = 0.15; see also Fig. 2F,G). Tactile patterns appearing with congruent visual patterns were associated with the best performance (2.98 ± 0.79), followed by unimodal patterns (2.6 ± 0.94) and incongruent stimulus pairs (2.04 ± 0.97). Post-hoc analysis including Bonferroni correction (dividing the significance level α by 3 resulted in a corrected α of 0.0167) showed that all stimulus levels (unimodal, congruent and incongruent) differed significantly from each other (paired-samples t-tests: unimodal vs. congruent: $t_{32}$ = − 4.84, *d* = − 0.84; unimodal vs. incongruent: $t_{32}$ = 6.28, *d* = 1.09; congruent vs. incongruent: $t_{32}$ = 9.14, *d* = 1.59; all *p*-values < 0.001). Post-hoc analysis of the interaction effect (including Bonferroni correction) showed that only the contrast of unimodal and congruent stimuli yielded significance between older and younger participants ($t_{31}$ = − 4.02, *p* < 0.001, *d* = − 1.4; Fig. 2G). In other words, older adults profited significantly more from crossmodal stimulus congruence in their detection performance compared to the younger group. Finally, tACS did not influence sensitivity measure d′.

The analysis of bias c showed a similar overall pattern: Main effects of AGE ($F_{1,31}$ = 15.4, *p* < 0.001, $\eta_{p}^{2}$ = 0.44; see Fig. 2H) and STIMULUS ($F_{2,62}$ = 11.4, *p* < 0.001, $\eta_{p}^{2}$ = 0.27; Fig. 2I) as well as a significant interaction of these two factors were observed ($F_{2,62}$ = 6.32, *p* = 0.005, $\eta_{p}^{2}$ = 0.17; Fig. 2J,K). The average bias for the older group was − 0.14 (SD 0.11), indicating a liberal response tendency, whereas the younger group obtained an average value of − 0.02 (SD 0.07; see also Fig. 2H). Interestingly, only the older participant group showed a response bias significantly different from zero ($t_{15}$ = –5.28, *p* < 0.001, *d* = − 1.32). Average biases across all participants were the following: − 0.08 ± 0.15 for the unimodal condition, − 0.15 ± 0.18 for congruent stimulus pairs and − 0.0008 ± 0.13 for incongruent pairs. Post-hoc tests revealed that the stimulus effect was driven by the difference between congruent and incongruent conditions ($t_{32}$ = − 4.62, *p* < 0.001, *d* = − 0.8; Fig. 2I) while all other contrasts were not significant. Of note, only the unimodal ($t_{32}$ = − 3.19, *p* = 0.003, *d* = − 0.56) and the congruent ($t_{32}$ = − 4.54, *p* < 0.001, *d* = − 0.79) conditions were associated with bias values significantly different from zero. Bias differed between older and younger participants between unimodal and congruent conditions ($t_{31}$ = 2.86, *p* = 0.008, *d* = 1), as well as between congruent and incongruent stimulus pairs ($t_{31}$ = − 3.87, *p* < 0.001, *d* = − 1.35; Fig. 2K). For both contrasts, differences between conditions were more pronounced for the older participant group. Finally, tACS did not influence bias c.

### Response times

CDFs were estimated as Gumbel functions using kernel density estimation^35^ on condition-pooled data. Pairwise comparisons reflecting main and interaction effects of all within- and between-participants factors of the mixed ANOVA design were performed by subtraction of CDFs. Non-parametric statistical evaluation was based on confidence intervals estimated by subtraction of shuffled data CDFs. Reported *p*-values reflect the minimum level of significance that needs to be exceeded along the whole range of RTs. Instead of exact *p*-values for the individual effects, we report RT ranges in which the *p*-value is undershot. Effect sizes were estimated for pairwise comparisons with Cohen’s *d* at the response time of the maximal group effect. Details of the statistical approach can be found in the “Methods” section.

**Figure 3:**
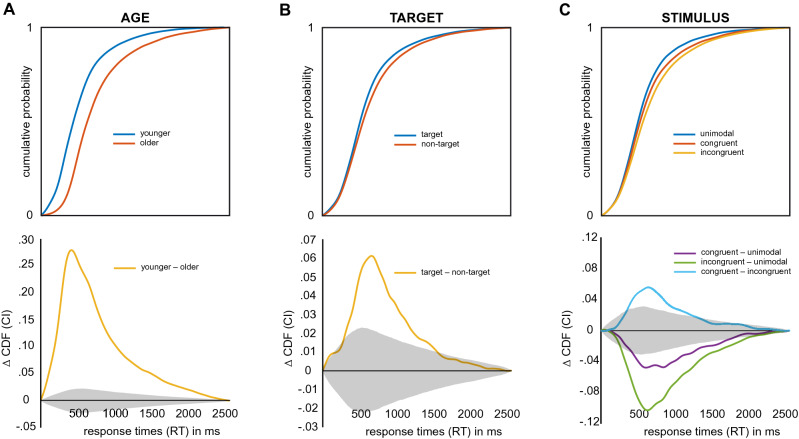
Main effects of AGE, TARGET and STIMULUS on cumulative distribution functions (CDFs) of response times (RTs). Grey shaded areas depict confidence intervals corrected for multiple testing. (**A**) (top) CDFs broken down by AGE. (bottom) Pairwise differences between AGE groups. (**B**) (top) CDFs broken down by TARGET. (bottom) Pairwise differences between TARGET conditions. (**C**) (top) CDFs broken down by STIMULUS. (bottom) Pairwise differences between STIMULUS conditions.

First, we found main effects for the factors AGE, TARGET and STIMULUS (Fig. 3). Overall, younger participants responded faster than older participants (Fig. 3A; CDF threshold: younger 787 ms, older 926 ms). This difference was significant across the whole range of RTs (*p* < 0.0013; younger-older: 0–2500 ms, *d* = 1.15 at 824 ms). Furthermore, target and non-target trials differed significantly in response speed. When the pattern to be detected was presented in the tactile channel (target), participants responded significantly faster compared to when a non-target would be presented as the tactile stimulus (Fig. 3B). This difference was significant for responses between 720 and 1720 ms (*p* < 0.00131, *d* = 0.78 at 1016 ms). Lastly, we found RT distributions to be significantly shaped by the nature of the stimulus. Unimodal stimuli showed overall fastest responses, congruent visuotactile stimuli were relatively delayed and incongruent visuotactile stimuli were slowest (Fig. 3C). All pairwise comparisons showed significant differences (*p* < 0.00039; unimodal-congruent: 830–1870 ms, *d* = 0.40 at 975 ms; unimodal-incongruent: 700–2120 ms, *d* = 0.95 at 993 ms; congruent-incongruent: 780–1540 ms, *d* = 0.77 at 1002 ms).

**Figure 4:**
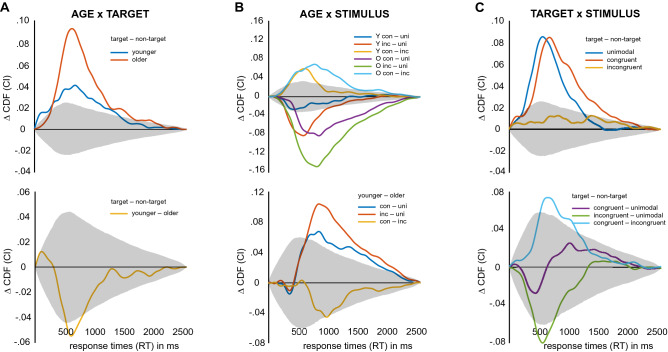
Interactions between experimental factors AGE, TARGET and STIMULUS on cumulative distribution functions (CDFs) of response times (RTs). Grey shaded areas depict confidence intervals corrected for multiple testing. (**A**) Interaction between AGE and TARGET. (top) Simple effects of TARGET separately for both AGE groups. (bottom) Differences between simple effects of TARGET. (**B**) Interaction between AGE and STIMULUS. (top) Simple effects of STIMULUS separately for both AGE groups. (bottom) Differences between simple effects of STIMULUS. (**C**) Interaction between TARGET and STIMULUS. (top) Simple effects of STIMULUS for both levels of TARGET. (bottom) Differences between simple effects of STIMULUS.

Secondly, the experimental factors interacted in shaping response times (Fig. 4). AGE affected the amount of both the TARGET and the STIMULUS effect. Although both age groups showed significantly faster responses on target trials (Fig. 4A top; *p* < 0.00065; younger: 0–1610 ms, older: 720–1820 ms and 1860–2090 ms), older participants showed a significantly larger positive effect of target presentation (Fig. 4A bottom; *p* < 0.00145; younger-older: 910–1140 ms, *d* = 0.64 at 977 ms). Significant stimulus effects were seen in both age groups for all but one comparison: in younger participants, unimodal and congruent trials did not differ with respect to response times (Fig. 4B top; *p* < 0.00018, younger unimodal-congruent: none, younger unimodal-incongruent: 700–1300 ms, younger congruent-incongruent: 770–1160 ms, older unimodal-congruent: 850–2260 ms, older unimodal-incongruent: 700–2370 ms, older congruent-incongruent: 740–2090 ms). When comparing these simple effects of stimulus condition across age groups, we found that the difference between unimodal and congruent as well as incongruent conditions was significantly stronger in older participants (Fig. 4B bottom; *p* < 0.00039, younger-older, unimodal-congruent: 1010–2160 ms, *d* = 1.21 at 1163 ms; younger-older, unimodal-incongruent: 990–2270 ms, *d* = 1.05 at 1165 ms). The contrast between congruent and incongruent conditions, however, did not show significant difference with respect to AGE (Fig. 4B bottom). Additionally, stimulus effects differed between target and non-target trials. That is, the beneficial effect of the target was not present for incongruent stimulus presentations (Fig. 4C top; *p* < 0.00039, unimodal: 670–1480 ms, congruent: 500–630 and 760–2130 ms, incongruent: none). Pairwise comparisons of the target effect showed that the effect’s magnitude was comparable between unimodal and congruent stimuli, but was significantly smaller in incongruent trials compared to both others (Fig. 4C bottom; *p* < 0.00039; unimodal-congruent: none; unimodal-incongruent: 830–1090 ms, *d* = 1.00 at 938 ms; congruent-incongruent: 910–1360 ms, *d* = 0.72 at 1004 ms).

**Figure 5:**
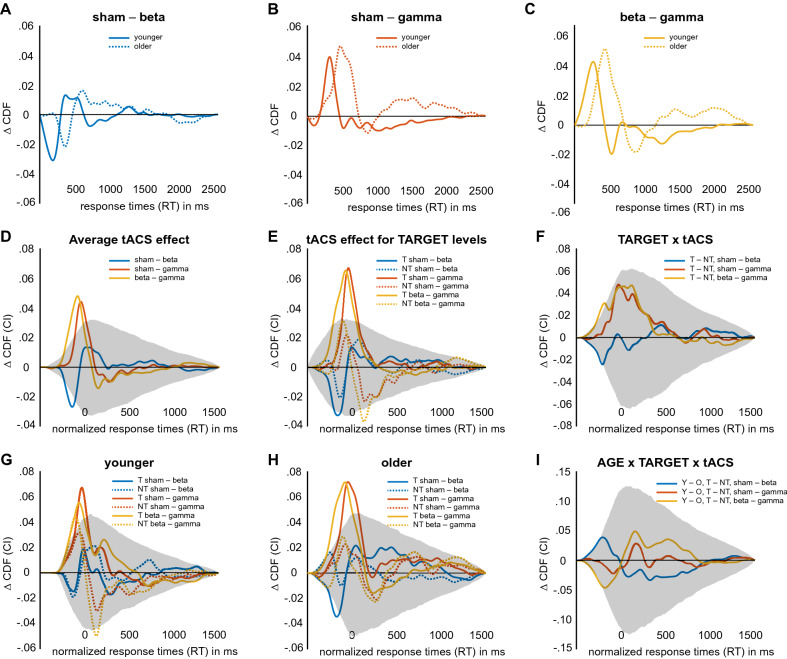
tACS-related differences between cumulative distribution functions (CDFs) of response times (RTs). Grey shaded areas depict confidence intervals corrected for multiple testing. (**A**) Effect of beta tACS (20 Hz) on uncorrected CDFs shown separately for age groups (same data with confidence intervals is depicted in Supplementary Fig. 1). (**B**) Effect of gamma tACS (70 Hz) on uncorrected CDFs shown separately for age groups. (**C**) Difference between beta and gamma tACS on uncorrected CDFs shown separately for age groups. (**D**) Average tACS effect on normalized CDFs. (**E**) tACS effect on normalized CDFs shown for target (solid lines) and non-target (dashed lines) trials. (**F**) Interaction effect between TARGET and tACS. (**G**) The same as in (**B**), but shown only for the younger group. (**H**) The same as in (**B**), but shown only for the older group. (**I**) Interaction between AGE, TARGET and tACS.

Finally, tACS influenced response times. Overall, we found that beta stimulation led to more fast responses and gamma stimulation to fewer fast responses, a result pattern that was highly comparable between age groups (Fig. 5A–C). This result can be seen as a within- study replication that supports the overall validity of the tACS effects. Yet, due to the strong main effect of AGE, tACS effects of the older group were shifted rightwards on the x axis. As a consequence, comparing tACS effects between age groups resulted in a marginally significant interaction (see Supplementary Fig. S1F). In order to account for the main effect of age, we normalized CDFs with the threshold of the age groups’ average CDF (Supplementary Fig. S1B,E; threshold younger: 787 ms, threshold older: 926 ms), resulting in a non-significant interaction between AGE group and tACS condition (Supplementary Fig. S1G). The tACS effect based on normalized CDFs is depicted in Fig. 5D (*p* < 0.00028). Under beta stimulation, the likelihood of fast responses between − 230 and − 110 ms relative to the group median was significantly increased compared with sham. Gamma stimulation, in contrast, led to a significant decrease of fast responses when compared with sham (− 110 to 10 ms relative to the group median). As a consequence, we found significant differences between beta and gamma stimulation for responses between − 230 and − 20 ms relative to the group median (*p* < 0.00028; sham-beta: *d* = 0.11 at − 142 ms; sham-gamma: *d* = 0.16 at − 46 ms; beta-gamma: *d* = 0.15 at − 82 ms). Importantly, we found that tACS effects were only significant for target trials (Fig. 5E,F). While the slowing of responses under gamma tACS was equally pronounced for younger and older participants, the speeding effect of beta tACS was significantly more pronounced in the older group (Fig. 5G–I).

### Relation between behavioural performance and cognitive status

**Figure 6:**
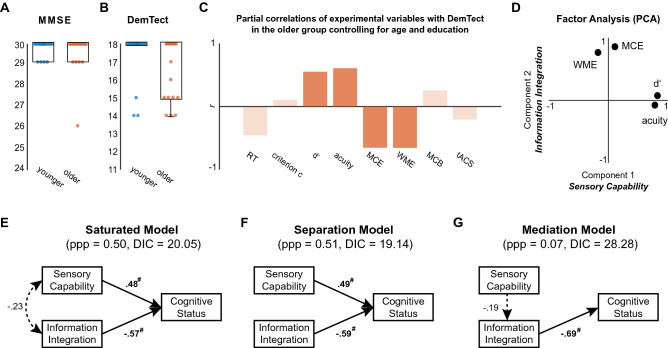
Bayesian structural equation modelling (BSEM) of cognitive status. (**A**) Distributions of Mini-Mental State Examination (MMSE) scores for younger and older participants. (**B**) Distributions of DemTect scores for younger and older participants. (**C**) Partial correlations between cognitive status (DemTect) and grand average response time (RT), grand average criterion c, grand average d′, average sensory acuity (acuity, see “Methods” for details), multisensory congruence enhancement (MCE, see “Methods” for details), working memory enhancement (WME, see “Methods” for details), multisensory congruence bias (MCB, see “Methods” for details) and the average tACS effect (tACS, see “Methods” for details). Correlations are controlled for the influence of age and education. Shading indicates non-significant correlations at *p* > 0.05. (**D**) Principal component analysis (PCA) separated the four variables into two distinct components termed “Sensory capability” and “Information integration”. (**E**) Path diagram of the saturated model. (**F**) Path diagram of the separation model. (**G**) Path diagram of the mediation model. Dotted lines show non-significant paths (# signifies that the Bayesian estimate’s credibility interval did not include 0). Standardized estimates are shown. *ppp *posterior predictive p value. *DIC *deviance information criterion.

In order to investigate links between task performance and cognitive status, we conducted a multi-level analysis including Bayesian structural equation modelling (BSEM). First, we assessed the distributions of DemTect and MMSE scores in both younger and older participants. For MMSE scores, both groups did not show significant deviation from the maximum score (both *p* = 0.062). For DemTect scores, only the older group showed significant deviation from the maximum score (*p* = 0.004), while the younger group did not (*p* = 0.250). Thus, DemTect but not MMSE was sensitive to variability of cognitive status in the older group in the absence of disease (Fig. 6A,B). This finding is in line with previous work that showed DemTect to have higher specificity and sensitivity than MMSE^36^.

Secondly, we explored pairwise partial correlations with DemTect controlling for age and education in order to identify relevant factors for BSEM. Reported statistics are not corrected for multiple comparisons. For this analysis, we considered the following variables (for details please see the “Methods” section): (i) grand mean RT, (ii) grand mean criterion c, (iii) grand mean d′, (iv) sensory acuity, a metric based on unimodal visual and tactile thresholds, (v) MCE (multisensory congruence enhancement), i.e. the maximal simple effect of crossmodal stimuli on d′ (congruent—unimodal), (vi) WME (working memory enhancement), a measure for the main effect of TARGET on RT, (vii) MCB (multisensory congruence bias), i.e. the maximal simple effect of crossmodal stimuli on criterion c (congruent—incongruent), and (viii) the mean tACS effect on RT. In younger adults, we did not find significant correlations of any of these variables with DemTect. These results, however, are difficult to interpret in the context of ceiling effects^37^. In older adults, we found DemTect to correlate positively with d′ (*r* = 0.61, *p* = 0.022) and sensory acuity (*r* = 0.62, *p* = 0.019) as well as negatively with MCE (*r* = − 0.7, *p* = 0.006) and WME (*r* = − 0.74, *p* = 0.002; see Fig. 6C and Supplementary Fig. S3 for scatter plots). Of note, these groups of variables correlated within (d′ ~ sensory acuity: *r* = 0.66, *p* = 0.010; MCE ~ WME: *r* = 0.66, *p* = 0.010; see Supplementary Fig. S2), but not significantly across groups (d′ ~ MCE: *r* = − 0.14, *p* = 0.632; d′ ~ WME: *r* = -0.53, *p* = 0.051; sensory acuity ~ MCE: *r* = − 0.27, *p* = 0.356; sensory acuity ~ WME: *r* = − 0.44, *p* = 0.119). No other significant correlations with DemTect were observed. A full description of all pairwise correlations between experimental variables in both age groups can be found in Supplementary Fig. S2 and the corresponding text.

Third, we investigated potential underlying factors of the observed variables with exploratory factor analysis (EFA) in the group of older adults. Due to the relationship between the two groups of variables (reflected in moderate correlations), we conducted a principal component analysis (PCA) with oblique rotation (Oblimin with Kaiser Normalization) on the residual data of the four variables d′, sensory acuity, MCE and WME. Established tests for the assumptions of this approach were conducted (see “Methods”)^38,39^. We were able to retain two components which jointly explained 84.02% of the variance. Factor loadings after rotation are shown in Table 2, for the pattern matrix as well as the structure matrix. As can also be seen in Fig. 6D, the four variables were resolved in two clearly separable components which showed insignificant, weak correlation (*r* = − 0.23, *p* = 0.395). In the following, we will refer to these two components with “sensory capability” (component 1, with high loadings for d′ and sensory acuity) and “information integration” (component 2, showing high loadings for MCE and WME), respectively.

**Table 2:** Summary of the exploratory factor analysis.

Variable	Rotated factor loadings	
	Component 1: “Sensory capability” (pattern matrix/structure matrix)	Component 2: “Information integration” (pattern matrix/structure matrix)
d′	0.93/0.91	0.07/− 0.14
Acuity	0.88/0.90	− 0.09/− 0.29
MCE	0.13/− 0.08	0.95/0.92
WME	− 0.19/− 0.38	0.85/0.89

Results from oblique rotation (Oblimin with Kaiser Normalization) for the pattern and the structure matrix are shown. The four variables were resolved in two dimensions, labelled “sensory capability” and “information integration”.

Finally, we completed a path analysis in the group of older adults based on the factor scores from the two components and DemTect to investigate how “sensory capability” and “information integration” relate to cognitive status. Specifically, we tested the saturated regression model with two predictors against two rival reduced models: (1) the “separation model” describing the hypothesis of separate (independent) explanatory factors for cognitive status, and (2) the “mediation model” describing the hypothesis of a mediating effect of “information integration” with “sensory capability” being the main driver (see also Fig. 6E–G). Using Bayesian estimation (for details see “Methods” section), the following posterior mean regression coefficients (standardized values) were observed for the saturated model (Fig. 6E): 0.48 for the influence of “sensory capability” on cognitive status and − 0.57 for the influence of “information integration”. Correspondingly, the 95% credibility intervals (CI) were [0.18 0.77] and [− 0.82 − 0.26] suggesting relevant effects (since 0 was not contained in the intervals). If orthogonality was assumed between “sensory capability” and “information integration” (“separation model” in Fig. 6F), the posterior mean regression coefficients changed slightly to 0.49 [0.17 0.76] and − 0.59 [− 0.83 − 0.25]. Again, we can be quite sure that both dimensions were relevant predictors of cognitive status. In contrast, the “mediation model” (Fig. 6G) showed “information integration” to explain cognitive status with a coefficient of − 0.69 [− 0.88 − 0.36] but the direct influence from “sensory capability” onto “information integration” was negligible: − 0.19 [− 0.61 0.31]. What is more, the indirect effect from “sensory capability” onto cognitive status (not shown in Fig. 6G) was insubstantial: 0.13 [− 0.22 0.47]. When comparing the reduced models on the basis of the posterior predictive p value (ppp^40^) and the deviance information criterion (DIC^41^), we found that the “separation model” (ppp = 0.51, DIC = 19.14) clearly outperformed the “mediation model” (ppp = 0.07, DIC = 28.28). In fact, only the “separation model’s” ppp suggested decent fit. The “separation model” even showed a tendency to be preferred over the saturated model (ppp = 0.5, DIC = 20.05).

### tACS-related adverse events

None of the participants reported severe or moderate adverse effects^42^ related to the electrical stimulation. Mild adverse effects^42^ were monitored using a custom-made questionnaire that quantified the intensity and time course of skin sensations, phosphenes and pain (see “Methods”). Older participants did not report significant mild adverse events related to stimulation (Supplementary Fig. S4C; median intensities: skin sensations 0 ± 0, phosphenes 0 ± 0, fatigue 0 ± 0, pain 0 ± 0; all *p* > 0.5). Younger participants reported skin sensations (1 ± 0.75, *p* = 0.001) and fatigue (1 ± 2, *p* = 0.004) but no phosphenes (0 ± 0, *p* = 0.5) or pain (0 ± 0, *p* = 0.25). None of the comparisons within groups were significant between conditions (all *p* > 0.1). Between-group comparisons showed that younger participants perceived significantly stronger skin sensations (*p* = 0.002) and fatigue (*p* = 0.026) compared with the older group (Supplementary Fig. S4A,C). We additionally asked participants to rate the time course of sensation. Ratings across all sensations were used to compute binary variables indicating sensations only in the beginning (0) or at any or all later time points (1; mean values younger: sham 0.24, beta 0.82, gamma 0.47; mean values older: sham 0.06, beta 0.31, gamma 0.13). Chi-square tests showed that younger participants were not significantly blinded for the difference between sham and beta-/gamma-frequency stimulation (*p* = 0.001), but comparisons between beta- and gamma-frequency were non-significant (Supplementary Fig. S4B). Older adults were blinded for all comparisons between sham, gamma- and beta-frequency stimulation (Supplementary Fig. S4D; all *p* > 0.2).

## Discussion

In our visuotactile match-to-sample task, irrelevant visual input delayed responses overall but showed differential effects on detection sensitivity of tactile patterns: while crossmodal incongruence of tactile and visual patterns was associated with diminished discrimination sensitivity, crossmodal congruence improved detection. Both costs and benefits of visuotactile interactions were more pronounced in the older group. At the same time, however, older participants showed a liberal response bias, which was absent in the younger group. Further, all participants showed speeded responses when the tactile target pattern was presented. Again, this effect was stronger in the older group. tACS with beta frequency (20 Hz) speeded responses whereas gamma stimulation (70 Hz) delayed responses. Strikingly, the speeding effect of beta-frequency stimulation was more pronounced in the older group, but only for target stimuli. Finally, Bayesian structural equation modelling (BSEM) suggested the existence of two largely independent factors explaining the cognitive status of healthy older adults: sensory capability and information integration.

Numerous studies have demonstrated that multisensory integration changes across the lifespan^11,12,43^. Our data adds to these findings and supports the hypothesis that ageing is characterized by enhanced multisensory integration. Specifically, older adults showed increased sensitivity in discriminating tactile dot patterns due to task-irrelevant but congruent visual co-stimulation. Notably, this effect of multisensory integration was present despite selective attention to the tactile input. Integration of attended information from one sensory modality with unattended input from another modality has been shown before^44,45^ and its enhancement in older adults is compatible with the recent proposal that ageing is associated with impaired weighing of crossmodal input^13^. According to this idea, younger adults weigh stimuli from different modalities according to their relevance, while older adults tend to integrate all available information^13^. Given that younger participants in our study did not show an effect of multisensory enhancement on sensitivity, our data supports this notion. Further, multisensory enhancement of tactile stimulus processing led to a levelling of discrimination sensitivity between younger and older adults. Potential for compensation of decreased sensory acuity by enhanced multisensory integration has been shown before^21^ and was suspected to follow directly from the degradation of sensory systems^11^. If this was the case, however, we should have observed a correlation between measures of sensory acuity and the extent of multisensory enhancement, which was not the case. Therefore, our data suggests that, while older participants can compensate for decreased sensitivity to tactile input through multisensory integration to some degree, enhanced integration in ageing does not simply follow from the degradation of sensory systems.

Importantly, however, we found a liberal response bias in the older group. Older adults showed a tendency to report detection of the target pattern even in its absence and thus produced more false alarms. Specifically, we found a liberal decision criterion in unimodal trials, which was enhanced for congruent crossmodal trials but absent for incongruent crossmodal trials. Similar results were reported by Mishra and Gazzaley^46^ who described enhanced multisensory sensitivity in older adults to be confounded by elevated false alarm rates in an audiovisual task. The authors contributed this finding to age-related deficits in inhibitory control^47^. Although there is a lack of studies investigating response tendencies as a function of age in crossmodal settings, a rich body of literature describes bias differences related to age in the context of recognition memory^48^. It has been proposed that these alterations in decisional processes result from false attribution of familiarity^49,50^ and behavioural studies support the idea of an age-related shift from recollection-based to familiarity-based mnemonic strategies^51,52^. Strikingly, familiarity in a task employing audiovisual stimuli has been shown to modulate activity within the same cortical regions as does crossmodal congruence^53^. We suggest that the absence of crossmodal conflict in our study evoked familiarity-based processes in older participants and thereby introduced a liberal response bias. It should be considered that modulations of response bias due to crossmodal congruence might be an overlooked finding in other studies that evaluated overall response accuracy. As a consequence, the magnitude of multisensory enhancements might have been overestimated in some cases.

In the current study, response times to crossmodal stimuli were overall delayed compared with unimodal stimuli. Slowing of responses was especially pronounced for crossmodally incongruent information, but also present when congruent visual patterns were presented^54^. This finding contrasts with studies showing speeded responses to redundant/congruent crossmodal stimuli in simple reaction time tasks^55,56^ or when evaluation of crossmodal congruence was explicitly instructed^17,57–60^. Yet, it is well compatible with studies that report crossmodal distraction in working memory paradigms^61,62^. In our task, tactile patterns had to be matched with a sample pattern held in working memory. Our data thus supports the notion that working memory processes are vulnerable to crossmodal distraction. Importantly, we show that this effect is enhanced in the older group, which is in line with earlier findings of increased distractibility of older adults in a visuotactile task^63^. Additionally, we report marked reductions in response latencies when matching between working memory content and tactile information was successful. This finding is in agreement with studies showing enhanced processing of sensory stimuli that match the content of working memory^64–66^. Notably, this effect was moderate for younger adults but strongly enhanced in the older group. Collectively, we present evidence for older adults’ increased crossmodal distractibility^67,68^ as well as their enhanced benefit from successful working memory matching.

As outlined above, we confirmed our hypotheses concerning enhanced multisensory integration in healthy older adults. In line with the recent proposal of impaired sensory weighing in ageing^13^, we found both enhanced costs and benefits of processing crossmodal stimuli in the older group. Our second research question asked how these indices of multisensory integration relate to overall sensory capability, and whether any or both of these measures would explain cognitive status. In the first step of our modelling approach, we identified four variables that significantly correlated with cognitive status in the older group: positive correlations for sensory acuity and d′, respectively negative correlations for the effect of working memory matching and the effect of multisensory enhancement with DemTect scores. These findings are noteworthy in two ways. First, we replicated earlier results suggesting that reductions in sensory acuity and cognitive status go hand in hand in ageing^7–9^. Second, we found an inversed relationship for multisensory enhancement. That is, older participants with lower cognitive status benefitted more from congruent crossmodal stimulation than older participants with maximum scores of cognitive status. In other words, those older participants who resembled the younger group in terms of cognitive status, also resembled the younger group in terms of multisensory enhancement.

While a number of studies investigated associations between multisensory integration and cognitive abilities in pathological conditions^69^, there is a lack of studies that address how multisensory integration and cognitive status relate during healthy ageing. One recent study that directly investigated this link did not find significant correlations between multisensory gains and measures of cognitive status in healthy older adults, but found a positive correlation for participants suffering from mild cognitive impairment (MCI)^70^. While it is possible that links between multisensory integration and cognitive status fundamentally differ between healthy and pathological ageing, differences between the aforementioned study and ours can help to elucidate the disparity of findings. First, the correlation reported in Murray et al. was based on MMSE scores, which we did not find to be sufficiently sensitive to variability of cognitive status in our analysis compared with DemTect^31^. Second, and in contrast to our paradigm, the authors measured response times in a simple audiovisual detection paradigm which neither involved the discrimination of complex spatial stimuli nor any working memory component. In comparison, our paradigm can be expected to involve more widespread cortical networks underlying more complex multisensory effects. It remains an open question how low-level perceptual and more complex effects of multisensory integration can be delineated and whether distinct mechanisms or at least different involvement of brain regions are underlying. Third, multisensory effects in Murray et al. depended on profiles of sensory dominance that were calculated as response time differences between auditory and visual trials using basic salient stimuli. Though incorporating individual sensory (dominance) profiles to investigate multisensory effects seems a promising avenue^71,72^, our study is fundamentally different as it investigates the identification of spatial patterns presented at individual sensory thresholds and their matching to working memory content. Future studies will need to further clarify how different experimental designs, task demands and involved sensory modalities influence multisensory integration in the context of ageing.

Based on the pattern of significant bivariate correlations of sensory acuity, d′, multisensory congruence enhancement (MCE) and working memory enhancement (WME) with cognitive status, we asked next how these variables related to each other. We found that the beneficial effect of working memory matching (reflected in WME) correlated with the beneficial effect of congruent crossmodal stimulation (MCE) in older adults, while neither of these effects depended on sensory acuity or grand mean d′, a general marker of discrimination performance. Factor analysis suggested that the latter measurements load onto a common component which we labelled ‘sensory capability’, whereas MCE and WME relate to a second factor which we called ‘information integration’. In a path analysis, we finally asked whether the influence of sensory capability and information integration on cognitive status is better described by separate causation, or by mediation of sensory capability via information integration. The latter model is motivated by a scenario where enhanced multisensory integration follows from degraded sensory processing. Model comparison showed clear evidence for the idea that sensory capability and information integration explain largely independent aspects of the cognitive status. Thereby, our data adds to the existing literature and shows that sensory decline and enhanced information integration can be independent and possibly represent two separate processes of ageing that in turn might result from distinct underlying neurophysiological processes.

While sensory decline can likely be mapped onto alterations in peripheral sensory organs and sensory cortices, underlying neural structures and processes of information integration are less clear. Inspecting the literature, a candidate structure that has been highlighted as a hub in multisensory as well as working memory networks is the posterior parietal cortex^25,72,73^. Studies with a wide range of methodological approaches have identified brain regions in the posterior parietal cortex, mainly including the inferior parietal sulcus (IPS), the inferior parietal lobule (IPL) and the superior parietal lobule (SPL), as a core network for multisensory integration processes^74–77^. Likewise, these regions have reliably been associated with tasks involving working memory^73,78–81^. As the enhancement of multisensory integration correlated with the enhancement of working memory matching for the older group in our study, giving eventually rise to the underlying construct we coined information integration, we speculate that an age-related enhanced tendency to integrate information might be associated with alterations in (cortical) networks centred on the posterior parietal cortex. Furthermore, since substantial evidence suggests that both multisensory integration and working memory matching are implemented by interactions of oscillatory activity in cortical networks^18,19,59,60,82^, we speculate that enhanced information integration might be related to altered oscillatory coupling.

This speculation is supported by our results related to the tACS manipulation. Compared with sham, 20 Hz tACS over left IPS and primary somatosensory cortex (S1) speeded responses while 70 Hz stimulation delayed responses. Importantly, this effect was more pronounced for target trials in which sensory information matched the memorized pattern in working memory. We propose that the putative boosting of beta power by tACS in this parieto-central network facilitated the reactivation of working memory content and its matching to the information processed in somatosensory cortex^83^. Gamma-frequency stimulation, on the other hand, might have had an overall inhibiting impact on beta oscillations and thereby hindered reactivation and/or working memory matching. This opposing nature of beta- and gamma-frequency tACS has been noted earlier^84–87^, and has been suggested to reflect cross-frequency modulations between gamma- and beta-band oscillations^87,88^. Finally, we found the interaction of tACS and working memory effects to be enhanced for older participants, affirming the high relevance of the parietal cortex’s integrative function in the older group. Taken together, we propose that older adults’ higher tendency of information integration might be related to altered oscillatory dynamics in the posterior parietal cortex.

Implications of our results are limited in a number of important ways. The sample size in the subgroup of healthy older adults was modest for our exploratory analysis approach including BSEM, demanding cautious interpretation of the findings. Keeping this general caveat in mind, we argue that many aspects of the simple models evaluated in our work, especially the high factor loadings, the low number of estimated parameters and the definition of informative prior distributions, favour the usage of BSEM^89^. It has been argued that in contrast to maximum likelihood (ML) estimation often used in classical frequentist SEMs, Bayesian estimation does not necessarily depend on large sample sizes^90,91^. We are convinced that the usage of Bayesian estimators and fit indices in our analysis including model comparison^90,91^ corroborates our conclusions. Nonetheless, future work is needed to substantiate our findings in older populations. The lack of structural magnetic resonance imaging (MRI) data prevented us from constructing individual head models in order to adapt tACS montages for optimal targeting of cortical sources^92^. Also, we designed montages based on a task-positive beta network found in an earlier study in young adults^19^ and, thus, did not account for potential age-related changes in location and frequency of the underlying networks. Yet, finding significant modulation by tACS that was largely comparable across age groups suggests that this approach can be successful. Nonetheless, individual tailoring of stimulation parameters will most likely reduce variance and elevate effect sizes. Finally, we did not record M/EEG and thus cannot detail on the functional underpinnings of the behavioural effects.

Taken together, we provide evidence corroborating the idea that healthy ageing is accompanied by enhanced processing of multisensory information. Importantly, we could show that the effects of enhanced multisensory integration were independent of unimodal sensory capability and are thus unlikely a consequence of sensory degradation. We argue that enhanced multisensory integration in older adults might reflect an overall enhanced tendency to integrate information that, in our data, was also reflected by behavioural benefits of successful working memory matching. We propose that the posterior parietal cortex could have an important role in mediating augmented integration during ageing, which is in line with a rich body of literature on overall parietal functions and supported by the effects of beta-frequency tACS found in this study. Importantly, behavioural indices of both sensory capability and information integration explained variability in the cognitive status of healthy older adults. As we found these groups of predictors to be independent, future work should be dedicated to delineate these possibly distinct processes of brain ageing.

## Methods

### Participants

We re-invited 13 younger and 15 older adults from a cohort that was enrolled in a previous behavioural study^20^ and additionally recruited new participants in order to include 17 individuals in each group. The mean time interval between the preceding study and the current study was 18.17 months (SD 1.07). In the older group, we excluded one re-invited participant due to a decline in the cognitive status (DemTect < 13). The final sample consisted of 17 younger (10 female, 24.77 ± 3.09 years) and 16 older (10 female, 72.56 ± 4.55 years) volunteers. All participants were right-handed according to the Edinburgh handedness inventory^93^, had age-adjusted normal or corrected-to-normal vision, no history or symptoms of neuro-psychiatric disorders and no history of centrally acting drug intake. All participants received monetary compensation for participation in the study. The study was conducted in accordance with the Declaration of Helsinki and was approved by the local ethics committee of the Medical Association of Hamburg (PV5085). All participants gave written informed consent.

### Assessment

Prior to the experiment, every participant underwent an assessment procedure consisting of a neurological examination, the Mini-Mental State Examination (MMSE)^30^ and the DemTect^31^ to rule out symptoms of neuro-psychiatric disorders and to examine the cognitive status. A 2-point-discrimination test^28^ was conducted to ensure intact peripheral somatosensation and a test of the visual acuity (Snellen chart)^29^ to ensure intact vision. If necessary, the assessment as well as the experimental procedure were performed with corrected vision. Additionally, every participant rated the subjectively experienced attention level, general fatigue and fatigue of the hand at the beginning of the experiment by placing a mark on a 10-cm visual analogue scale (VAS). The extremes of the horizontally positioned VAS were labelled: attentive vs. inattentive, awake vs. tired, hand not tired vs. hand tired. The VAS values were analysed by measuring the position of the mark in cm.

### Experimental procedure

After the assessment, we prepared an EEG cap in which electrodes for the electric stimulation were mounted and applied an analgesic creme (see “tACS stimulation” for details). In a training session, participants got acquainted with the experimental setup, the stimulus material and experimental task. After the four dot patterns (Fig. 1B) were introduced visually and tactilely, participants were trained to match two consecutively presented tactile dot patterns. Training blocks of 24 pairs had to be completed until matching accuracy was at 75% correct or above (min. 3 blocks, max. 10 blocks). The experiment consisted of three main blocks of 17.5 min duration in which tACS was applied with sham, beta-frequency (20 Hz) or gamma-frequency (70 Hz) stimulation. The sequence of tACS conditions was counterbalanced across participants. At the beginning of each block, tACS was ramped up before experimental trials began (see “tACS stimulation” for details). Each block consisted of 192 trials, split in four sessions of 48 trials separated by short breaks of 30 s. After completion of each of the three main blocks, we asked participants to complete a questionnaire assessing the side-effects of the electric stimulation. Taken together, participants completed 576 trials in about 60 min.

### Experimental design

Participants’ task was to compare tactile patterns to a target pattern and report match or mismatch as fast and accurately as possible via button press (see also “Experimental setup”). The target pattern was chosen pseudo-randomly from the stimulus set (see Fig. 1B) and was newly defined for each of the four sessions within a block (so each of the four patterns served as target in every tACS block). Each trial started with a central fixation of 1000 ms, followed by the presentation of a unimodal tactile or crossmodal visuotactile stimulus for 500 ms (Fig. 1A). The tactile pattern was administered via a Braille stimulator (see “Stimulus material” for details), either unimodally (1/3 of all trials), or accompanied by a congruent (1/3) or incongruent (1/3) visual stimulus presented on a computer screen. The response interval was limited to 2750 ms, after which feedback was given by colouring the fixation cross green or red. In half of all trials in a given session of 48 trials, we presented the target pattern as the tactile stimulus. Overall, each block contained equally many stimuli of each condition (unimodal, congruent and incongruent) and featured all four patterns as targets in pseudo-randomized order.

### Experimental setup

The experiment took place in a light-attenuated chamber and participants were comfortably seated in a chair with their right hand resting on a custom-made board containing the Braille stimulator (QuaeroSys Medical Devices, Schotten, Germany). Tactile patterns were administered to the participants’ right index fingertip while visual patterns were presented on a 21-inch computer screen running at 60 Hz with a resolution of 1024 × 768 pixels, positioned 110 cm in front of the participants. Eye position was monitored continuously to ensure that participants kept central fixation. Participants responded using their left middle or index finger (counterbalanced within both age groups) to press one of two buttons on a response box (Cedrus, Model RB-834, San Pedro, USA). We used Presentation software (Neurobehavioral Systems, Version 16.3) to control stimulus presentation and to record participants’ response times (RTs) and accuracies.

### Stimulus material

The Braille stimulation cell consisted of eight independently controllable pins arranged in a four-by-two matrix, each pin 1 mm in diameter with a spacing of 2.5 mm. The set of tactile stimuli used in the current experiment included four geometric patterns (see Fig. 1B), each of them formed by four elevated pins. Visual stimuli were designed analogously to the tactile patterns and presented left of a central fixation cross on a noisy background (Perlin noise; Fig. 1A). The visual patterns subtended 3.5° × 2.5° of visual angle. To achieve comparable performance in pattern detection in older and younger participants, tactile and visual stimulus intensities were adjusted individually. We matched the amplitude of pin elevation as well as the grey intensity of the visual patterns to individual thresholds^20^. The Braille stimulator used allowed the pin elevation to be controlled in 4095 discrete steps, with a maximum amplitude of 1.5 mm. Maximal grey intensity equalled black (RGB: 0-0-0). For participants who already participated in the preceding study, we used the originally estimated thresholds. The validity of the estimated thresholds was checked for all participants in the training session prior to the actual experiment. Except for the one excluded older participant, all other older participants still performed at the accuracies defined during the preceding study at their individual thresholds. The newly recruited participants underwent the thresholding procedure one week prior to the experimental session. For the detailed description of the adaptive staircase procedure see^20^.

### tACS stimulation

Multi-electrode transcranial alternating current stimulation (tACS) was administered via two separate stimulators (DC-Stimulator Plus, Neuroconn, Germany) used in external mode. That means, current output was precisely controlled via voltage input to the stimulator that was produced by a NI-DAQ device run with Labview (NI USB 6343, National Instruments, USA). For each montage, we used five Ag/AgCl ring electrodes (diameter = 12 mm) organised in 4-in-1 montages. These montages aim to restrict current flow under the central electrode and thereby increase focality of stimulation^94^. To that end, electrodes were prepared such that impedances were homogenous within montages and as low as possible (below 50 kΩ). Cortical targets were chosen from a previous study that identified a left-hemispheric beta-band network to be associated with target detection in a similar paradigm as employed here^19^. In accordance with these results, we prepared montages over left primary somatosensory cortex (S1) and left intraparietal sulcus (IPS) and simulated resulting electric fields. Current densities were estimated by using the inverse model constructed by means of exact low-resolution electromagnetic tomography (eLORETA)^95^ on the basis of a boundary-element three-shell head model^96^ in combination with a cortical grid (MNI152). The resulting electric field at location $\vec{x}$ was then modelled as the sum of linear combinations between eLORETA leadfield ($\vec{L}$) and injected currents at the stimulation electrodes ($\alpha_{i}$):

1 $$\vec{E}\left( {\vec{x}} \right) = \mathop \sum \limits_{i} \left( {\vec{L}\left( {\vec{x}} \right)\alpha_{i} } \right).$$

In order to compensate for differences in the anatomy of the skull (thickness, curvature, etc.) that impact the strength and depth of resulting electric fields, we determined the necessary amount of electric current to be administered over S1 and IPS in order to achieve current densities of up to 0.3 V/m in the targeted cortical regions. As a consequence, we applied 1.68 mA peak-to-peak over S1 and 2.4 mA peak-to-peak over IPS. Stimulation current was ramped up from 0 to maximal intensity within 10 s prior to the experimental blocks and afterwards delivered constantly with either 20 Hz (beta) or 70 Hz (gamma). In sham blocks, stimulation was terminated after the ramp-in. In order to minimize transcutaneous side-effects of stimulation, we used a creme of eutectic mixture of local anaesthetics (EMLA) applied to the skin under each ring electrode. Side-effects were tracked with a questionnaire after each tACS block.

### Statistical analyses

Statistical analyses were performed in Matlab (Version 9.1, MathWorks, Natick, MA, 2014) using custom-made scripts and IBM SPSS AMOS (Version 21.0.0, Build 1178, IBM, 2012).

#### Assessment data

To test for group differences in the assessment data a multivariate analysis of variance (MANOVA, R’s *manova()* command) was performed with the values for 2-point-discrimination, visual acuity, tactile threshold, visual threshold, MMSE, DemTect, fatigue, education level, attention level and fatigue of hand as dependent variables and group (younger participants vs. older participants) as the independent variable. For post-hoc analysis, (univariate) ANOVAs were performed. As the results of the assessment parameters are not independent, Benjamini–Yekutieli correction^97^ was used to adjust for multiple comparisons.

#### Detection accuracy

Detection performance was analysed using measures provided by signal detection theory (SDT)^32,33^, namely sensitivity d′ and criterion location c (or bias). Whereas sensitivity captures the perceiver’s ability to discriminate different choice alternatives (e.g., targets vs. non-targets or, more general, signal vs. noise), response bias describes the perceiver’s tendency to categorize stimuli as either the one or the other^98^, regardless of actual stimulus presence. In the case of target detection, bias statistics reflects the degree to which “yes” or “no” choices are favoured^33^. Both d’ and c are calculated from hit and false alarm rates. In our paradigm, we defined hits and false alarms for the different conditions as follows: hits were equated with correctly identified tactile targets, either presented without a synchronous visual pattern (*unimodal*), or in combination with a *congruent* respectively an *incongruent* one. False alarms in contrast are usually defined as 1 − correct rejections, in our case 1 − the ratio of correctly identified non-targets, again either appearing in their *unimodal*, *congruent* or *incongruent* version. For these three conditions, we computed d′ values by subtracting the z-transformed false alarm (FA) rates from the z-transformed hit (H) rates, within both older and younger adults and for the three tACS conditions separately:

2 $$d^{\prime} = z\left( H \right) - z\left( {FA} \right).$$

In the framework of SDT, bias measure c corresponds to the position of the decision criterion relative to the point of intersection between the (internal) distributions of signal and noise (or two signals). As in yes/no detection tasks, we computed c as:

3 $$c = - {\raise0.7ex\hbox{$1$} \!\mathord{\left/ {\vphantom {1 2}}\right.\kern-\nulldelimiterspace} \!\lower0.7ex\hbox{$2$}}\left( {z\left( H \right) + z\left( {FA} \right)} \right).$$

Response bias can be described as liberal (c < 0, corresponding to a tendency to say “yes” or in our case to (over-) detect targets), conservative (c > 0, corresponding to a tendency to detect non-targets) or neutral (c = 0, not systematically favouring either of the choice alternatives). In the terminology of SDT, a negative criterion in our study reflects a liberal bias to report targets for both trials where targets really are presented (hits) as well as non-target trials (false alarms). Analogously, a positive criterion value points to a conservative bias and a tendency to report non-targets in trials actually containing targets (misses) as well as in non-target trials (correct rejections). Lastly, a neutral bias is present if false alarm and miss rates are equal. To analyse task performance in our crossmodal detection paradigm, we subjected d’ as well as c values to a mixed repeated-measures ANOVA design with the between-participants factor AGE (younger/older) as well as the within-participants factors STIMULUS (unimodal/congruent/incongruent) and TACS (sham/beta/gamma). In addition, bias was tested against zero to check whether there would be any statistically significant response tendency at all.

#### Response times

Response times (RTs) were analysed on the level of distributions. The shape of RT distributions between 500 and 2500 ms after stimulus presentation was estimated with Gaussian kernels^35^ and compared between conditions as cumulative distribution functions (CDFs). Statistical evaluation was performed with permutation statistics for the complete factorial design of *AGE* (younger/older) × TARGET (target/non-target) × STIMULUS (unimodal/congruent/incongruent) × TACS (sham/beta/gamma). To that end, we shuffled single-trial data across conditions and participants in order to form N collections of RT data, where N corresponds to the maximal number of collections for computing a given contrast. For instance, estimating the null distribution for the evaluation of TARGET, we iteratively build two collections from the shuffled RT data, computed CDFs for each collection and subtracted the CDFs. The null distribution for each contrast was computed by storing the CDF differences of each permutation (100,000 permutations). Confidence intervals for each main factor and interaction were built by finding percentiles according to *alpha* and 100-*alpha*. Initially, *alpha* was set to 2.5/n %, where n is the number of minimal multiple comparisons of a given factor/interaction. This *alpha* accounts for both the two-tailed analysis and the multiple comparisons due to the factorial design, but not for the range of RTs tested. In other words, the type I error is controlled for locally at each RT bin, but not globally for the whole distribution. This was achieved by increasing percentiles until maximally *alpha* % of all null-differences would exceed the CI globally. The resulting CI controls type I error globally at *alpha*. The actual condition differences were then computed in a similar fashion by first pooling data across all participants and sub-conditions. CDFs were computed on the pooled data and subtracted according to the factorial design. Differences exceeding the CI at any point of the RT range were treated as a significant condition difference.

#### Bayesian structural equation modelling (BSEM) of cognitive status

Path modelling of causal relations between experimental variables and cognitive status was performed with SPSS^99^ and AMOS^100^ in four steps:

1. Choice of variable(s) for the estimation of the cognitive status was based on the statistical evaluation of potential ceiling effects, which were expected at least for the younger group. We computed signed rank tests for deviation from maximum score for both measures (MMSE: 30, DemTect: 18). Data of group and measurement was only used when there was a significant deviation from the maximum, i.e. absence of ceiling effect (*p* < 0.05).
2. We pooled data into eight variables reflecting overall behavioural traits or central behavioural effects. For the former category, we used grand average response time (RT), grand average criterion c as a measure of response bias (criterion c), grand average d′ as a measure of perceptual sensitivity (d′) and averaged perceptual thresholds as a measure of overall sensory acuity (acuity). Prior to averaging, data was z transformed.
  4 $$acuity = - {\raise0.7ex\hbox{$1$} \!\mathord{\left/ {\vphantom {1 2}}\right.\kern-\nulldelimiterspace} \!\lower0.7ex\hbox{$2$}}\left( {z\left( {threshold_{tactile} } \right) + z\left( {threshold_{visual} } \right)} \right)$$

As central behavioural effects, we added a compound measure of multisensory interactions referred to as multisensory congruence enhancement (MCE)^17,101^, a compound measure of the main TARGET effect on RT labelled working memory enhancement (WME) as well as a measure of multisensory congruence bias (MCB). For the calculation of MCE and MCB, we chose the contrast yielding maximal effect size in the ANOVA post-hoc analysis in the older group.

5 $$MCE = d^{\prime}_{congruent} - d^{\prime}_{unimodal}$$

6 $$WME = RT_{nontarget} - RT_{target}$$

7 $$MCB = criterion \hspace{1mm} c_{congruent} - criterion \hspace{1mm} c_{incongruent}$$

8 $$tACS = RT_{beta} - RT_{gamma}$$

For the computation of the tACS effect, we identified the latency of the maximal effect on group level (largest positive deflection of the yellow curve in Fig. 5D) and computed individual CDFs and respective contrasts per participant. We considered the value of this contrast at the group level latency as the magnitude of the tACS effect for a given participant.

In order to identify relevant factors for the path modelling, we computed bivariate partial correlations between these variables and cognitive status while controlling for the impact of age and education (cut-off: *p* < 0.05, uncorrected).

(3) Dimensionality of the data was reduced by means of exploratory factor analysis (EFA) using principal component analysis (PCA) with oblique rotation (Oblimin with Kaiser Normalization) on the residual data of the four variables d′, sensory acuity, MCE and WME. Using oblique rotation accounted for the observed weak to moderate (anti-) correlations between all combinations of variables. Sampling adequacy was verified using the Kaiser–Meyer–Olkin measure (KMO = 0.555). Additionally, all anti-image correlations for individual items were > 0.5. Bartlett’s test of sphericity indicated that correlations between items were sufficiently large for PCA (*χ*^2^ = 16.854, *p* = 0.01).
(4) Path modelling using Bayesian estimation was employed to evaluate two rivalling hypotheses: (1) Two separate (i.e. independent) factors (“information integration” and “sensory capability”) explain cognitive status (“separation model”), and (2) Mediating factors explain cognitive status, i.e. “sensory capability” influences cognitive status via “information integration” (“mediation model”). Further, these two models were compared to the saturated model (all possible paths are modelled). To estimate parameters of the three models, 500 samples were used for the burn-in period. Convergence of the posterior summaries was assessed based on a measure suggested by Gelman et al. (Gelman-Rubin statistic^40^). By default, AMOS judges the sampling procedure to have converged if the largest of the convergence statistic (C.S.) values is smaller than 1.002. For parameters estimated in our models, we specified the following prior distributions: normal distributions with a mean of 0 and a standard deviation of 1 (0,1) for regression parameters (weights and means), normal distribution (0,1) for the covariance of “sensory capability” and “information integration”, normal distributions (1,1) for the variances, normal distribution (0,1) for the intercept of “information integration” in the “mediation model”, and uniform distribution from 13 to 18 for the intercept of DemTect. The latter values reflect the restricted range of raw scores that were considered in our analysis. Model fit was evaluated based on the posterior predictive p value (ppp^102^) and the deviance information criterion (DIC^41^). Whereas ppp values around 0.50 indicate good model fit and small values misfit, the model with the smallest DIC among a set of competing models is preferred^91^.

#### tACS-related adverse events

The questionnaire tested the perceived maximum intensity as well as the time course of the following sensations: skin sensations (itching, warmth, stinging, pulsating), phosphenes, fatigue and pain (ranked as either “absent”/0, “light”/1, “moderate”/2, “pronounced”/3 or “strong”/4). The time course was evaluated as “beginning”, “end”, “always”. Differences in sensation intensity between age groups as well as between experimental conditions were evaluated with Wilcoxon signed rank tests for zero median. The effectiveness of blinding was assessed by comparing the time course of sensation between sham and verum (beta and gamma) stimulation. To that end, we computed binary compound scores across all sensation qualities that reflected whether participants rather perceived stimulation only in the beginning (0) or at any or all later time points (1). These binary scores were evaluated for significant differences between sham and verum using McNemar tests.

## Supplementary Information

Supplementary Information.

## Data Availability

Behavioural data will be made available upon request to the corresponding authors.
