# Supplementary material for: Sensory capability and information integration independently explain the cognitive status of healthy older adults
Source: Sci Rep. 2020 Dec 31;10:22437. doi: 10.1038/s41598-020-80069-8 (PMC7775431; doi:10.1038/s41598-020-80069-8)
Supplement: Supplementary file 1 — Supplementary Information. [file 41598_2020_80069_MOESM1_ESM.pdf]

Sensory capability and information integration independently explain the cognitive status of healthy older adults

Jonas Misselhorn[1#*], Florian Göschl[1#*], Focko L. Higgen[2#*], Friedhelm C. Hummel[3,4,5], Christian Gerloff[2§], Andreas K. Engel[1§]

[1]Department of Neurophysiology and Pathophysiology, University Medical Center Hamburg-Eppendorf, 20246 Hamburg, Germany
[2]Department of Neurology, University Medical Center Hamburg-Eppendorf, 20246 Hamburg, Germany
[3]Defitech Chair of Clinical Neuroengineering, Center for Neuroprosthetics and Brain Mind Institute, Swiss Federal Institute of Technology (EPFL), Geneva, Switzerland
[4]Defitech Chair of Clinical Neuroengineering, Center for Neuroprosthetics and Brain Mind Institute, Swiss Federal Institute of Technology Valais (EPFL Valais), Clinique Romande de Réadaptation, Sion, Switzerland
[5]Clinical Neuroscience, Medical School University of Geneva, Geneva, Switzerland

[*]Correspondence: j.misselhorn@uke.de, f.goeschl@uke.de, f.higgen@uke.de
[#]shared first authorship
[§]shared last authorship

Acknowledgements

This work was funded by the German Research Foundation (DFG) and the Natural Science Foundation of China (NSFC) in projects SFB TRR169/A3/B1/B4 and by the German Research Foundation (DFG) in projects SFB 936/A3/C1 and SPP 1665/EN 533/13-1.

Author contributions

JM, FG, and FLH designed the experiment. FCH, CG and AKE co-designed the experiment and provided funding. JM and FLH recorded the data. JM and FG analysed the data. JM and FG wrote the main manuscript text. JM, FG, FLH, FCH, CG and AKE reviewed the manuscript.

Competing Interests

The authors declare that they have no competing interests.

Data Availability

Behavioural data will be made available upon request to the corresponding authors.

Supplementary material

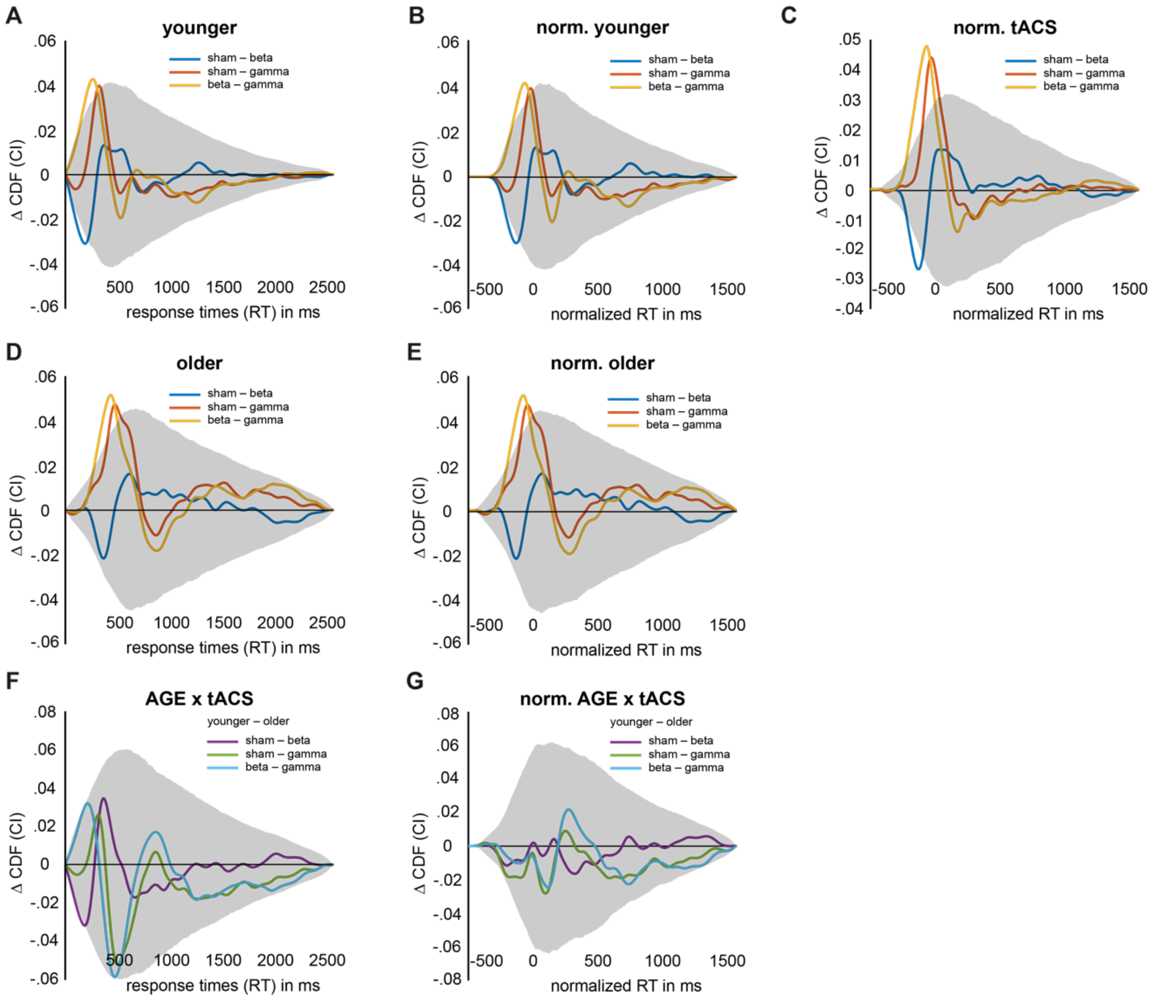

Figure S1. Interaction between transcranial alternating current stimulation (tACS) and AGE. Gray shaded area depicts corrected confidence interval. **(A)** Simple effects of tACS in younger participants. **(B)** Simple effects of tACS in younger participants for normalized cumulative distribution functions (CDFs). **(C)** Main effect of tACS for normalized CDFs. **(D)** Simple effects of tACS in older participants. **(E)** Simple effects of tACS in older participants for normalized CDFs. **(F)** Interaction effect between AGE and tACS. **(G)** Interaction effect between AGE and tACS for normalized CDFs.

Younger and older adults showed highly comparable result patterns when comparing response time (RT) distributions across tACS conditions (Figure S1 A,D). Due to a shift in RTs towards slower responses in the older group, however, the interaction between age and tACS showed marginal significance (Figure S1 F). In order to correct for this age-related latency difference, we subtracted all individual RTs with the group grand average RT. The analysis was then repeated on these normalized RTs (Figure S1 B,E) and no indication for an age x tACS interaction (Figure S1 G) was found. Simple tACS effects on the normalized RTs now show clear and distinct effects of stimulation (Figure S1 C).

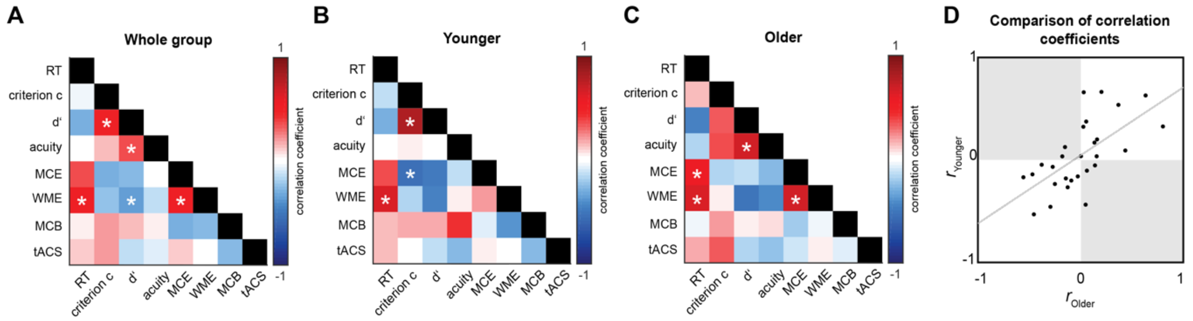

Figure S2. Partial correlations between experimental variables controlled for age and education. (A) Pairwise Spearman correlations for data of all participants (N=33). Asterisks indicate significance for p < 0.05. No correction for multiple comparisons was applied. **(B)** Pairwise Spearman correlations for data of the younger group (N=17). Asterisks indicate significance for p < 0.05. No correction for multiple comparisons was applied. **(C)** Pairwise Spearman correlations for data of the older group (N=16). Asterisks indicate significance for p < 0.05. No correction for multiple comparisons was applied. **(D)** Comparison of correlation coefficients across groups. Dots indicate data from (B) scattered against data from (C). Line represents least squares line. White tiles are areas of concordant correlation coefficients (same sign of r), whereas gray tiles indicate areas of discordant correlations (different sign of r). Abbreviations: RT…response time, criterion c…bias, d'…sensitivity, MCE…multisensory congruence enhancement, WME…working memory enhancement, MCB…multisensory congruence bias, tACS…transcranial alternating current stimulation (effect on RT).

The main behavioural measures response time (RT), bias measure criterion c, sensitivity d', multisensory congruence enhancement (MCE), working memory enhancement (WME), multisensory congruence bias (MCB) and the effect of transcranial alternating current stimulation (tACS) on RT as well as the outcome of the psychometric thresholding procedure preceding the experiment (acuity) are shown as a function of pairwise partial correlations controlling for age and education. Please be referred to the *Results/Methods* sections for explanation/definition of these variables. The assessment here is meant to support and expand the modelling approach in the main manuscript where we selected variables based on correlations with cognitive status (DemTect). To illustrate how all variables assessed in the experiment relate to each other and how these patterns differ between age groups, we computed correlations based on all participants (Figure S2 A), and based on the younger (Figure S2 B) respectively older (Figure S2 C) group. Please note that we did not correct for multiple comparisons, and thus, results should be understood as a descriptive appraisal of the data.

Overall, we find a significant positive correlation between WME and RT (r = 0.503, p = 0.004) that is present in both subgroups (younger: r = 0.633, p = 0.011; older: r = 0.630, p = 0.016). Additionally, the older group shows a significant correlation between the multisensory effect (MCE) and grand average RT (r = 0.537, p = 0.048). A significant positive correlation between criterion c and d' was found for the whole group (r = 0.471, p = 0.008) and the younger sub-group (r = 0.802, p < 0.001), but not in the older group (r = 0.327, p = 0.254). This age-related difference might be due to the overall significant bias in the older group which is absent in the younger group. Additionally, criterion c was negatively correlated with MCE in the younger group (r = -0.557, p = 0.031). The interpretation of the correlation findings involving bias is complicated by the fact that criterion c was found to be not significantly different from zero in the younger group. In the whole group as well as the older group, we found positive correlations between acuity and d' (whole: r = 0.368, p = 0.042; older: r = 0.660, p = 0.010), as well as between MCE and WME (whole: r = 0.515, p = 0.003; older: r = 0.664, p = 0.010). The correlation in the older group was replicated in the exploratory factor analysis (EFA) using principal component analysis (PCA) preceding Bayesian structural equation modeling (BSEM). In the younger group, we did not find such correlations indicating possible age-related associations between these measures (acuity ~ d': p = 0.914, MCE ~ WME: p = 0.468). Finally, we found a negative correlation between d' and WME for the whole group (r = 0.365, p =

0.044), but for neither sub-group. The subgroup correlation coefficients were both negative but not significant (younger: $r = -0.455$, $p = 0.090$; older: $r = -0.531$, $p = 0.051$).

Finally, we assessed the overall similarity of correlation patterns between age groups by computing a concordance measure that captures the proportion of correlation coefficients with the same sign across groups. Similarity was high with a concordance of 82%. Additionally, the correlation of correlation coefficients of younger and older adults was highly significant ($r = 0.638$, $p = 0.0003$).

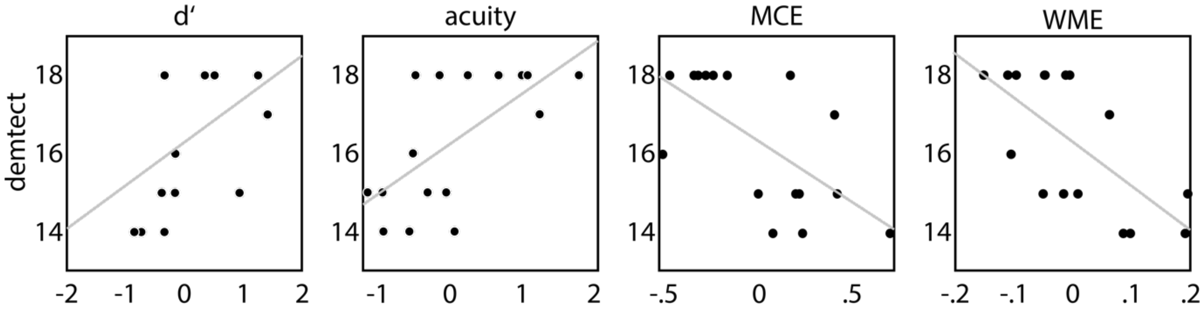

Figure S3. Bivariate partial correlations between cognitive status and experimental variables in older participants. Scatter plots show significant correlations from Figure 6 B. In order to correctly depict the partialisation for age and education, we plotted DemTect scores against the regression residuals of the respective variables. Abbreviations: MCE…multisensory congruence enhancement, WME…working memory enhancement.

DemTect ~ d': $r = 0.61$ $p = 0.022$
DemTect ~ sensory acuity: $r = 0.62$ $p = 0.019$
DemTect ~ MCE: $r = -0.70$ $p = 0.006$
DemTect ~ WME: $r = -0.74$ $p = 0.002$

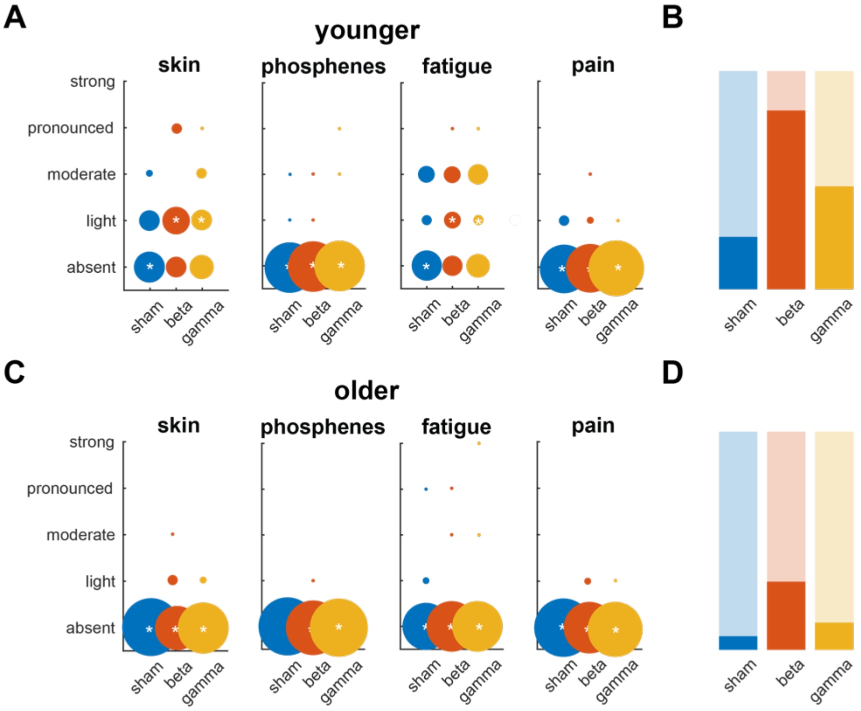

Figure S4. Questionnaire data of transcranial alternating current stimulation (tACS) side effects shown separately for skin sensations, phosphenes, fatigue and pain. (A, C) Younger (A) and older (C) participants rated maximum intensity of a given sensation for each stimulation block (sham, beta and gamma) from absent to strong. The size of circles indicates count of rating and median rating is marked with an asterisk. **(B, D)** Younger (B) and older (D) participants rated whether sensations were present only in the beginning (darker shade) or at any/all later timepoints in the blocks (lighter shade).
